# In Vivo Antischistosomicidal and Immunomodulatory Effects of Dietary Supplementation with *Taraxacum officinale*

**DOI:** 10.3390/jox14030056

**Published:** 2024-07-29

**Authors:** Amany Ebrahim Nofal, Amal Mohamed Shaaban, Hany Mohammed Ibrahim, Faten Abouelmagd, Azza Hassan Mohamed

**Affiliations:** 1 Histology and Histochemistry Unit, Zoology Department, Faculty of Science, Menoufia University, Shebin El-Kom 32511, Egypt; 2 Zoology Department, Faculty of Science, Menoufia University, Shebin El-Kom 32511, Egypt; amalosama708@yahoo.com (A.M.S.); azza_hassan_2006@yahoo.com (A.H.M.); 3 Immunology Unit, Zoology Department, Faculty of Science, Menoufia University, Shebin El-Kom 32511, Egypt; hany.mohamed@science.menofia.edu.eg; 4 Department of Medical Parasitology, Faculty of Medicine, Sohag University, Sohag 82524, Egypt; faten.rashad@gmail.com

**Keywords:** dandelion, fibrosis, granuloma, healing, SEM, schistosomiasis

## Abstract

Bilharziasis is a widespread trematode parasite that poses a severe public health burden. Dandelion (*Taraxacum officinale*) has several pharmacological and traditional properties critical for treating several hepatic disorders. The present study was designed to assess the potential efficacy of *T. officinale* root (TOR) dietary supplementation with or without praziquantel (PZQ) against liver and intestinal disorders in mice infected with *Schistosoma mansoni*. This study was conducted on five groups; G1: uninfected control, G2: untreated *S. mansoni*-infected mice, G3: infected animals treated with 250 mg/kg PZQ for three alternative days, G4: infected animals were orally administered 600 mg/kg bw TOR daily for 10 days, and G5: infected animals that received both PZQ and TOR as previously described. The current findings after different treatments indicated topographical scanning electron microscopy alterations of male adult worms and a critical reduction in worm burden, ova count, granuloma diameter, hepatic and intestinal histological abnormalities, fibrosis, immunohistochemical expression of CD3^+^ and CD20^+^ cells, oxidative stress, and interleukin-10, also upregulation of interferon-gamma, and antioxidant enzymes, when compared to the infected untreated mice. The best results were obtained in mice administered PZQ+TOR together because of their antioxidant properties and ability to promote the host immune response to parasitic infection.

## 1. Introduction

**Figure 1:**
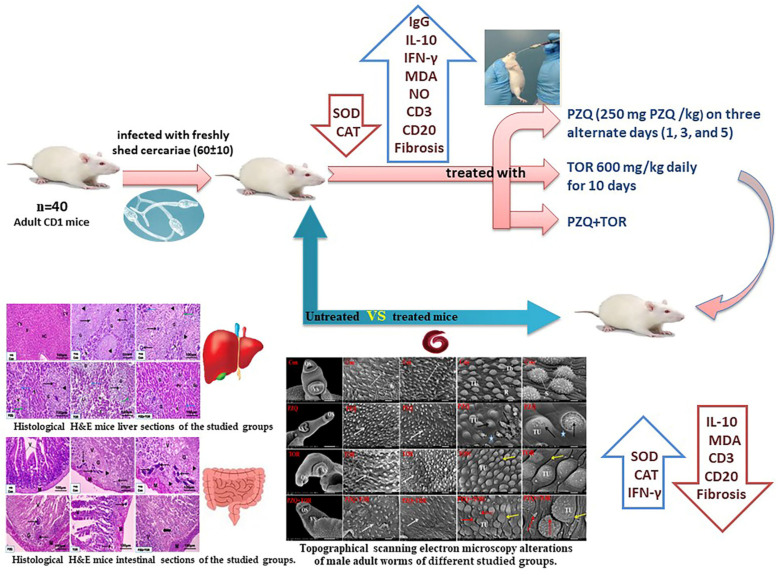
Schematic diagram of the study design.

To date, bilharziasis remains one of the most serious worldwide health issues caused by *Schistosoma* that threatens human life in many countries [1,2]. *Schistosoma mansoni* is one of the main neglected tropical waterborne diseases (NTDs) due to several environmental factors and behaviors that encourage snail survival in freshwater in Africa [3]. The World Health Organization (WHO) estimated that intestinal schistosomiasis (*S. mansoni*) affects at least 251.4 million people globally in Africa, the Caribbean, the Middle East, Venezuela, Brazil, and Suriname, and approximately 90% of them live in Africa [4]. Bilharziasis is the most common endemic parasitic illness in the world, notably in Egypt, and is one of the most common fibrotic conditions due to inflammatory reactions and scar tissue deposition surrounding parasitic eggs trapped in different organs, leading to several histological disorders, especially in the liver [5,6]. Most histopathological disorders in *Schistosoma*-infected animals have been attributed to immune host reactions to parasitic eggs caused by antigenic material [7]. Fibrosis and inflammation are common gastrointestinal disorders resulting from immune response stimulation due to granuloma formation and scar tissue around the parasite’s eggs [8]. As a result, the WHO targets bilharziasis elimination by 2030 according to the NTD roadmap [9]. The Egyptian government has taken comprehensive measures, such as hygiene education, overall chemotherapy, people behavior, snail control, and environmental changes, to effectively reduce the spread of infection and morbidity but is unfortunately unable to prevent reinfection [10]. To date, bilharziasis chemotherapy remains based on praziquantel (PZQ), a worldwide drug known to be effective against all species that cause bilharziasis, but it often incompletely ends parasitic infection due to its inactivity against young worms [1,11]. The long-term use of PZQ in combination with multiple treatments has led to the emergence of drug-resistant *Schistosoma* strains, so PZQ is unsuitable for therapy in endemic areas [12,13]. As a result of these problems associated with PZQ, the need to search for a new natural product, especially a medicinal plant, has been urgently promoted. Consistent with this assumption, Dkhil et al. [12], El-Hawary et al. [5], Shaaban et al. [14], and Abera [3] demonstrated that several plants have beneficial therapeutic effects, including anti-inflammatory, antioxidant, antifibrotic, and immunomodulatory effects, on experimental bilharziasis. The present study focused on a dietary supplement derived from medicinal plants rich in flavonoid and polyphenolic compounds. Dandelion, *Taraxacum officinale*, is a traditional herbal medicine from the *Asteraceae* family. Its whole plant, leaves, and roots are used to treat several gastrointestinal, cardiovascular, and gonad disorders [15,16,17]. However, little is known about its impact on intestinal schistosomiasis using intensive studies of topographical SEM alterations in male adult worms, oxidative stress, and the host immune response. Therefore, this study investigated the possible effect of *T. officinale* root (TOR) supplement, as a nutritional supplement with or without PZQ, on hepatic and intestinal disorders in *S. mansoni* infected mice (Figure 1).

## 2. Materials and Methods

### 2.1. Drugs and Dietary Supplements

Praziquantel tablets (PZQ, Epiquantel) were purchased from EIPICO (Ramadan City, Egypt), the Egyptian International Pharmaceutical Industries Company. The following dietary supplement product was used: Dandelion root 500 mg-100 Veg capsules, UPC: 733739046451; each capsule contained 500 mg dandelion root powder, Now Foods, 395 S, Glen Ellyn Rd, Bloomingdale, IL, USA.

### 2.2. Phytochemical and Antioxidant Assay of TOR

Total phenolics and flavonoids, as well as the radical scavenging activity of TOR against stable 2,2 diphenyl 2 picryl hydrazyl hydrate (DPPH), were estimated using the slightly modified methods of Barros et al. [18] and Padmanabhan and Jangle [19], respectively.

### 2.3. Animals

Healthy male CD1 strain albino mice (22 ± 5 g) and laboratory-bred snails (*Biomphalaria alexandrina*) infected with *S. mansoni* miracidia were obtained from Theodor Bilharz Research Institute (TBRI) in Giza, Egypt, for the present study. One week before the initiation of the experiment, the animals were acclimatized to laboratory conditions; they were housed in plastic rodent cages with a standard diet and ad libitum water at the animal facility of the Zoology Dept. after permission from the Institutional Animal Ethical Committee, Menoufia University (Approval ID: MUFS/S/Pa/1/22).

### 2.4. In Vivo S. mansoni Infection

After 4 weeks of snail infection, the snails were kept in dechlorinated tap water and exposed to artificial light at 28–30 °C for 2 h to stimulate cercaria shedding [20]. Each mouse was exposed to freshly shed cercariae (60 ± 10) [10].

### 2.5. Experimental Design

Forty male albino mice were utilized in the present work (eight mice per each group). Uninfected healthy mice (G1, were utilized as normal control). Mice were infected with 60 ± 10 freshly shed cercariae of *S. mansoni* without any therapy (G2, infected untreated group). After 7 weeks of infection, the infected mice were either treated orally with a freshly prepared aqueous suspension of PZQ (250 mg PZQ/kg) on three alternate days (1, 3, and 5) (G3, PZQ mono treatment) [21], orally administered 600 mg/kg bw TOR daily for 10 days (G4; TOR, monotherapy) [22], or orally administered both PZQ+ TOR at the previously mentioned doses in G3 and G4 (G5; PZQ+TOR, dual therapy).

Mice from all groups were euthanized with thiopental sodium for dissection at the end of the experiment, 2 months. Blood samples were collected in centrifuge tubes with or without anticoagulant for subsequent biochemical assays in plasma and serum. Liver and intestinal tissue samples were fixed in 10% buffered neutral formalin for paraffin block preparation for histopathological and immunohistochemical studies [23].

### 2.6. Parasitological Analysis

Perfusion of the mesenteric and portal veins was performed to retrieve worms from all mice for worm burden estimation [24]. Under a stereomicroscope (LW Scientific, Lawrenceville, GA, USA), the *S. mansoni* worms were classified according to sex and counted. The percentage reduction in the worm burden (%) in all groups was calculated as described by Osman et al. [21]. Small liver and intestinal pieces, approximately 0.5 g, were weighed, dried on filter paper and then placed in a glass test tube containing 5 mL of 4% KOH and potassium hydroxide solution for tissue digestion. Ova counts per gram of liver or intestine were determined as previously described [25]. The oogram patterns at different stages of ova development (immature, mature, and dead), maturity, and viability of approximately 300 ova eggs were investigated [26]. Adult male *S. mansoni* worms were obtained from infected untreated mice, and subsequently, the different current therapies were processed for examination by scanning electron microscopy (SEM) [27]. Adult male worms were scanned by SEM (Joel JSM-5300, Tokyo, Japan) at the Faculty of Science, Alexandria University, Egypt. The magnified areas of the adult male worms, especially the tubercles on the tegument and suckers, were photographed.

### 2.7. Histological Examinations and Granuloma Measurement

Paraffin sections of the liver and intestine (4–5 µm) were cut using a rotary microtome and stained with Ehrlich’s hematoxylin-eosin (H. & E.) [28] and Masson trichrome staining [29]. Histological sections were photographed by using an Olympus BX 41 microscope (Japan), and an ocular micrometer was utilized for measuring the granuloma diameter in a low-power field (×10, LPF), according to Jacobs et al. [30].

### 2.8. Cellular Immune Response

#### 2.8.1. Immunohistochemistry Examinations

On charged slices, hepatic and intestinal paraffin sections from all the mice were reacted specifically with rabbit monoclonal antibodies against CD3^+^ (cluster of differentiation 3 for T lymphocytes) and CD20^+^ (cluster of differentiation 20 for B lymphocytes) (Cell Marque, Rocklin, CA, USA), following the manufacturer’s instructions [31]. Positive brown staining was quantified by the Image J -win64 program (NIH, Bethesda, MD, USA) [32].

#### 2.8.2. Subtyping of Immune Cells

Fluorescent-labeled antibodies were used to measure CD4^+^ and CD8^+^ cells in the blood, and mononuclear blood cells were prepared at a density of one million cells/mL in RPMI-1640. A fluorescence-activated cell sorting flow cytometer (FACS, Becton Dickinson, Sunnyvale, CA, USA) was utilized. Anti-CD4^+^ and anti-CD8^+^ mouse monoclonal antibodies conjugated with fluorescein isothiocyanate (FITC; BD Biosciences, San Jose, CA, USA) were used. The data were analyzed using the Cell Quest program [33].

### 2.9. Biochemical Evaluation

Levels of total immunoglobulin G (T. IgG), interleukin 10 (IL-10), and interferon-gamma (IFN-γ) in blood samples were evaluated in serum by enzyme-linked immunosorbent assays (ELISAs) following the manufacturer’s instructions. Total IgG was determined using a mouse IgG ELISA Kit (Cat. No. E99-13, Bethyl Laboratories at Biomol, Montgomery, TX, USA). The mouse IL-10 concentration was determined by an ELISA kit sandwich (Thermo-Scientific, Boston, MA, USA). Mouse IFN-γ levels were quantified using a mouse IFN-γ ELISA Kit (Cat. No. RAF022R-BioVendor, Asheville, NC, USA). The level of the product of lipid peroxidation (malondialdehyde; MDA) was measured by the thiobarbituric acid assay [34]. Nitric oxide (NO) levels were determined following the method described by Montgomery and Dymock [35]. The activity of catalase (CAT) [36] and superoxide dismutase (SOD) [37] was estimated by a commercial kit (ZellBio GmbH, Lonsee, Germany).

### 2.10. Statistical Analysis

The collected data were presented as the mean ± standard deviation (mean ± SD). One-way analysis of variance (ANOVA) was used, followed by a post hoc test to determine the significance of differences between different groups. SPSS version 22.0 was used to detect the least significant differences (LSD), and *p* < 0.05 indicated statistical significance.

## 3. Results

### 3.1. Dandelion Root Supplement Phytochemical Composition and Antioxidant Activity

The total phenolic and flavonoid contents were quantified to estimate the phytochemical profile of the dandelion root supplement. The results of the phytochemical test revealed that the supplement was a good source of total phenolic compounds, with 13.14 ± 0.04 mg/g (mg gallic acid equivalents (GAEs) per 1 g of the supplement sample), and flavonoid compounds, with 3.96 ± 0.11 mg/g (mg quercetin equivalents (QEs) per 1 g of the supplement sample). The antioxidant activity of the dandelion supplement displayed strong scavenging effects (67.32 ± 0.22% inhibition) in the DPPH free radical assay. Overall, dandelion root supplements displayed relatively strong antioxidant activity at a relatively high percentage, which indicates better scavenging activity.

### 3.2. Parasitological Status

#### 3.2.1. Worm Burden, Ova Count, and Oogram Pattern

The current data in Table 1a–c revealed a significant (*p* < 0.05) decrease in the total worm burden, egg load, and oogram pattern changes, and an increase in the percentage of dead ova in the liver and intestines of all treated groups (PZQ, TOR, and PZQ+TOR) compared with infected untreated mice. Compared with those in untreated mice (G2), the monotherapies of PZQ alone (G3) and TOR alone (G4) resulted in a significant (*p* < 0.05) decrease in the total worm burden (with reduction percentages of 60.64% and 63.64%, respectively), hepatic ova count (with reduction percentages of 81.21% and 72.52%, respectively), intestinal egg load (with reduction percentages of 85.31% and 68.05%, respectively), and mature eggs in the liver and intestine, associated with a significantly increased count of dead ova in hepatic and intestinal tissues. However, no significant differences were detected in the mean numbers of immature eggs in hepatic and intestinal segments between mice that received PZQ or TOR alone (G3 and G4) and infected mice that did not receive therapy (G2). On the other hand, compared with G2, dual therapy with PZQ+TOR (G5) significantly reduced the total worm burden (69.10% reduction) and liver and intestinal ova count (81.71% and 86.86%, respectively). In addition, there was a significant (*p* < 0.05) increase in hepatic and intestinal dead ova counts and a significant (*p* < 0.05) decrease in liver and intestinal immature eggs in infected mice mono-treated with PZQ (G2). Oral administration of TOR alone or in combination with PZQ reduced the mean number of mature eggs in the hepatic and intestinal segments compared to that in the infected untreated mice.

#### 3.2.2. Topography of the Adult Male Worm

**Figure 2:**
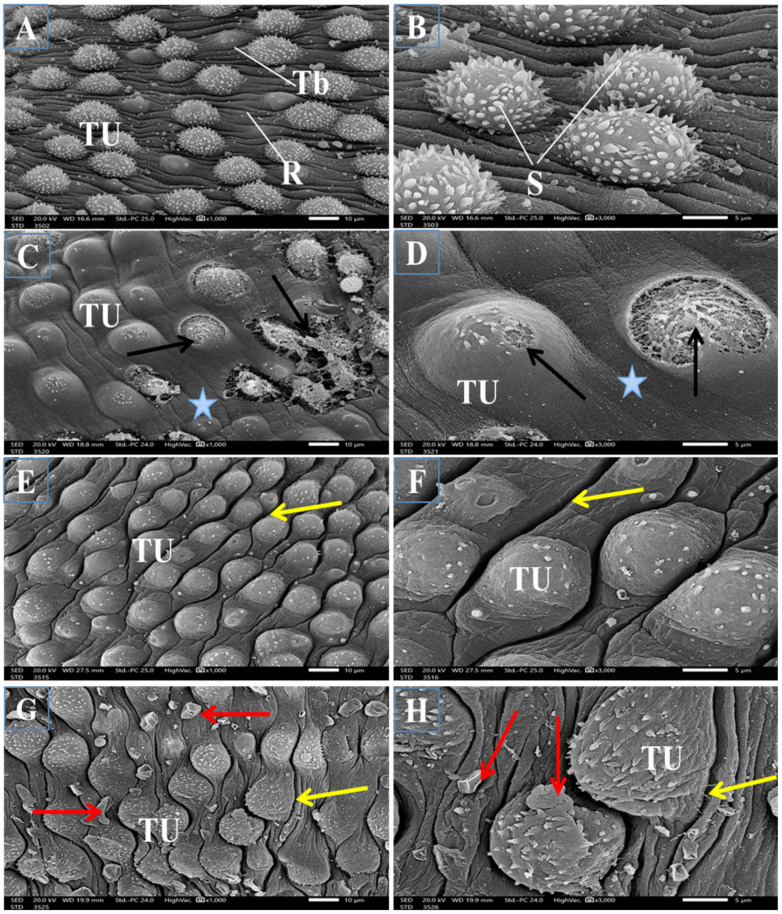
Representative scanning electron micrographs of the dorsal surface of adult male *S. mansoni* worms of the different groups (n = 3). (**A**,**B**): the worm from an infected untreated mouse has a rough dorsal tegument with numerous large tubercles (TU) bearing sharp spines (S) with small tegumental blebs (Tb) and tegumental ridges between them (R). (**C**,**D**): worm from the infected group treated with PZQ alone exhibiting worms with marked destruction of the tegument, erosion of the sloughing surface, swollen tubercles (TU) with a proximal loss of spines, extensive ulceration of the outer surface (black arrows), and loss of intertubercular tegumental ridges (stars). (**E**,**F**): the worm from an infected mouse supplemented with TOR alone showing distortion of the tubercles with loss of spines and wrinkling in the areas between them (yellow arrows). (**G**,**H**): the worm from PZQ+TOR-treated group exhibiting tegumental wrinkles (yellow arrows), distorted tubercles with markedly decreased spines, and many surrounding blebs (red arrows).

**Figure 3:**
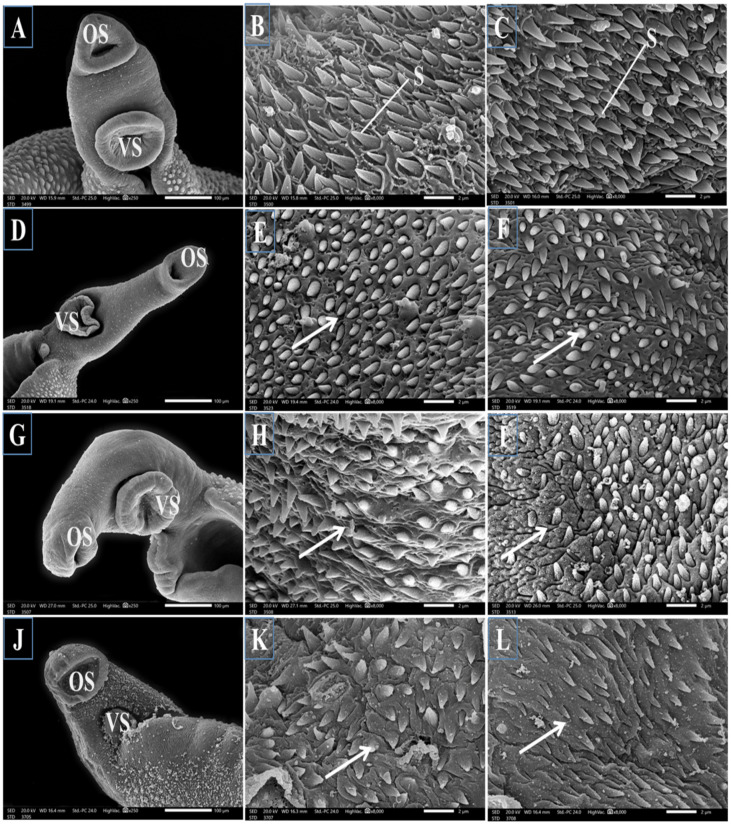
Representative scanning electron micrographs of the anterior region of adult male *S. mansoni* worms showing oral sucker (OS) and ventral sucker (VS) of the different groups (n = 3). (**A**–**C**): the worm from an infected untreated mouse showing normal morphology with oral and ventral suckers, respectively, with numerous sharp spines (S). (**D**–**F**) the worm from an infected mouse treated with PZQ exhibiting abnormal oral and ventral suckers with marked disturbances in the direction of the spines and a decrease in the number of spines (arrows). (**G**–**I**) the worm from an infected mouse supplemented with TOR displaying damaged oral and ventral suckers that lost their normal structure with marked disturbance in the direction of spines and a marked decrease in spines (arrows). (**J**–**L**) the worm from an infected mouse dual treated with both PZQ and TOR showing abnormal appearance of oral and ventral suckers with a marked decrease in the number of spines (arrows).

SEM examination of the outer surface of adult male *Schistosoma* worms from the infected untreated mice revealed a moderately rough dorsal tegument and ventral and oral suckers. The dorsal tegument is characterized by numerous large tubercles with tiny projections of concentrated directed spines and tegumental ridges with small blebs between them (Figure 2A,B). Normal ventral and oral suckers covered with numerous sharp spines varying in size were observed in the anterior region of the worm, with numerous sharp spines, rows of minute spines, and small blebs around the tubercles (Figure 3A–C, respectively). Male worms obtained from the PZQ mono-treated group displayed marked tegumental destruction, including erosion of the sloughing surface, swollen tubercles with a noticeable reduction in number, a proximally complete loss of spines, extensive ulceration of the outer surface, and loss of intertubercular tegumental ridges between them (Figure 2C,D). Moreover, obvious reductions in the number of spines on the oral and ventral sucker were observed (Figure 3D–F, respectively). The worms that were only treated with TOR exhibited a significant distortion of the tegument and collapsed tubercles characterized by the loss of spines with shrinkage and wrinkling in the areas between them (Figure 2E,F) with a noticeable decrease in spines in the oral and ventral suckers (Figure 3G–I, respectively). The worms dual therapy with PZQ accompanied by TOR exhibited tegumental wrinkles, complete disappearance of intertubercular ridges, and distorted tubercles with a noticeable reduction in their spines and many surrounding blebs (Figure 2G,H), with a marked decrease in the number of spines on the oral and ventral suckers (Figure 3J–L).

### 3.3. Histological Features

**Figure 4:**
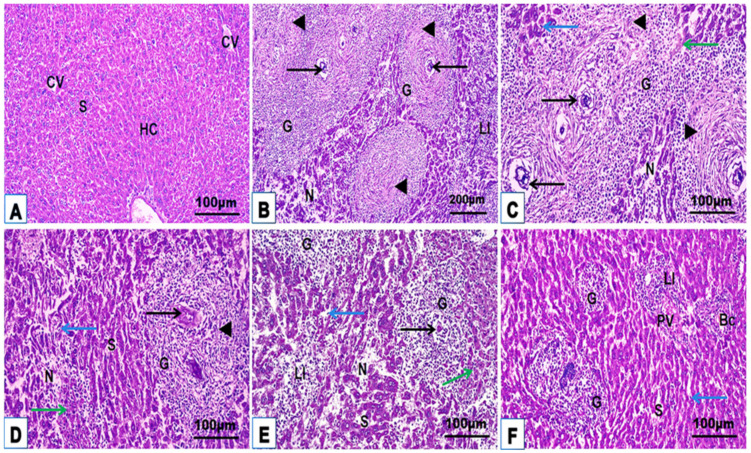
Representative photomicrographs from H. & E. mice liver sections of the studied groups (n = 5): uninfected control (**A**), infected untreated (**B**,**C**), PZQ-monotherapy (**D**), TOR-monotherapy (**E**), PZQ+TOR-dual therapy (**F**), demonstrating central vein (CV), hepatocytes (HC), sinusoids (S), cellular infiltration around the granuloma (G) with trapped parasitic ova (thin arrows), fiber deposition (black arrowheads), hypereosinophilic hepatocyte (green arrows), hypertrophied Kupffer cell (blue arrows), necrotic area (N), leucocytic infiltration (LI), portal vein (PV), and bile canaliculi (Bc) (H. & E. stain).

**Figure 5:**
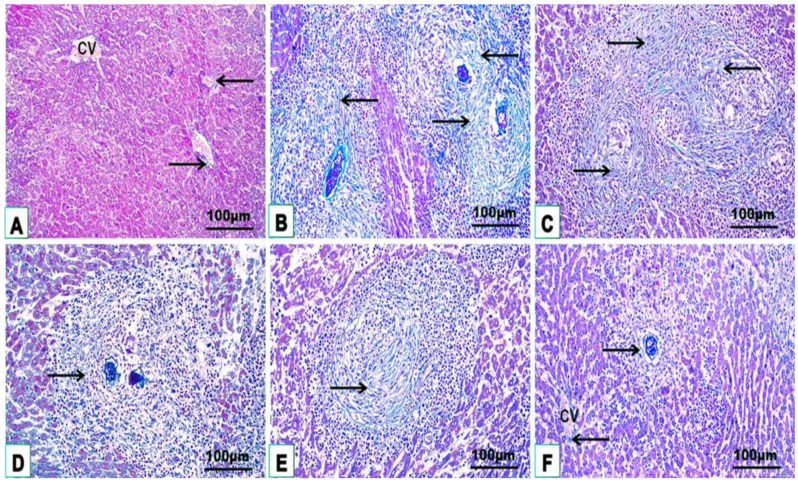
Representative photomicrographs from Masson trichrome mice liver sections of the studied groups (n = 5): uninfected control group (**A**), infected untreated group (**B**,**C**), PZQ-treated group (**D**), TOR-treated group (**E**), PZQ+TOR-dual therapy group (**F**), demonstrating blue–green collagen fibers distribution (arrows), and central vein (CV) (Masson’s trichrome stain).

Microscopic examination of histological hepatic H. & E. and Masson trichrome stained sections of uninfected control mice (G1) displayed a classical hepatic structure with organized strands of hepatocytes, central vein, portal tract, sinusoids with typical Kupffer cells, and little collagen fiber distribution around the blood vessels and sinusoids (Figure 4A and Figure 5A; Table 2). Liver sections from mice infected with *S. mansoni* without treatment (G2) revealed numerous multigranulomas of varying sizes and an average diameter of 152.93 ± 37.43 μm (Figure 4B,C; Table 2). The hepatic tissue surrounding these granulomas displayed marked loss of the normal hepatic architecture with severe histopathological changes characterized by hepatic strand disorganization with vacuolar degenerate and hypereosinophilic hepatocytes, dilated sinusoids with hypertrophied Kupffer cells, necrotic areas with karyolysis, inflammatory cell aggregation between hepatocytes and around the portal area, and dilated central and portal vein congestion (Figure 4B,C). These large granulomas were marked by one or more *Schistosoma* ova, causing severe chronic granulomatous inflammation surrounded by dense concentric fibrosis with numerous hepatic stellate cells, fibroblasts, macrophages, lymphocytes, and eosinophilic granulocytes (Figure 5B,C; Table 2). Monotherapy of infected mice with PZQ or TOR alone (G3 and G4) showed a mild to moderate degree of improvement characterized by few and small fibrocellular granulomas (90.14 ± 17.83 μm and 80.56 ± 21.19 μm, respectively), most of which were associated with dead ova, a significant reduction in inflammatory cells encircling the trapped ova, mild dilatation of blood sinusoids with hypertrophic Kupffer cells between slight vacuolar hypereosinophilic hepatocytes, and moderate collagen fibrous reactions around them compared with those in the infected untreated group (Figure 4D,E and Figure 5D,E; Table 2). Liver sections of infected mice after dual treatment (PZQ+TOR) (G5) showed a near-normal hepatic structure with significantly reduced granuloma diameters (38.97 ± 10.25 μm), causing a mild inflammatory response, including slightly dilated sinusoids with hypertrophic Kupffer cells, cellular infiltration around the empty positions devoid of eggs, and a portal area with semi-normal collagen fiber deposition (Figure 4F and Figure 5F; Table 2).

**Figure 6:**
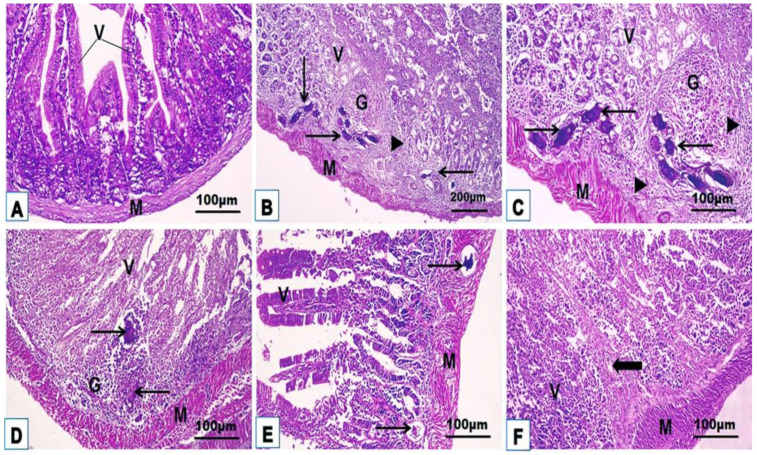
Representative photomicrographs from H. & E. mice small intestinal sections of the studied groups (n = 5): uninfected control (**A**), infected untreated (**B**,**C**), PZQ-monotherapy (**D**), TOR-monotherapy (**E**), PZQ+TOR-dual therapy (**F**), showing that the muscularis (M) and villi (V) extended from the submucosa to the lumen, cellular infiltration around the granuloma (G) with trapped parasitic ova (thin arrows), fiber deposition (black arrowheads), and cellular infiltration around the empty positions devoid of eggs (thick arrow) (H. & E. stain).

**Figure 7:**
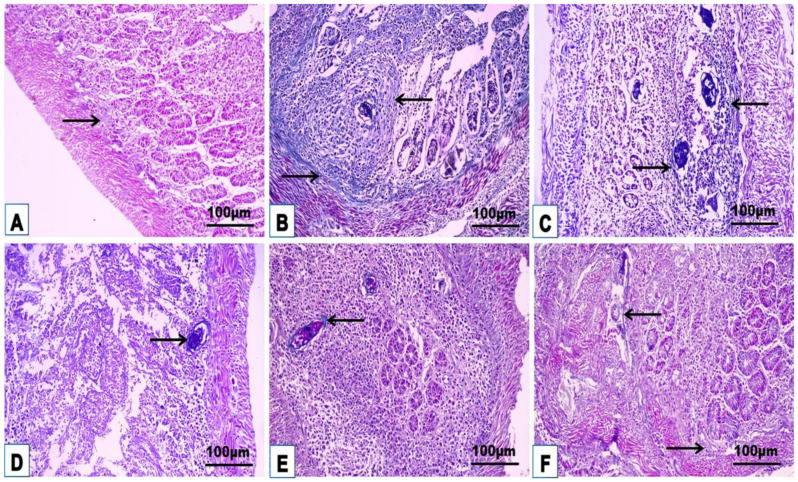
Representative photomicrographs from Masson trichrome mice small intestinal sections of the studied groups (n = 5): uninfected control (**A**), infected untreated (**B**,**C**), PZQ-monotherapy (**D**), TOR-monotherapy (**E**), and PZQ+TOR-dual therapy (**F**), demonstrating blue–green collagen fibers distribution (arrows) (Masson’s trichrome stain).

The intestinal H. & E. and Masson trichrome sections of uninfected control mice (G1) showed a proximal typical small intestinal histoarchitecture of the four well-organized general tunics (mucosa characterized by villi, submucosa, external muscularis, and outer serosa) with normal collagen fiber distribution in the lamina propria without any abnormalities (Figure 6A and Figure 7A; Table 2). Small intestinal sections from infected untreated mice (G2) exhibited a chronic inflammatory response against several large granulomas of varying sizes and an average diameter of 62.62 ± 2.08 μm in the mucosa and submucosa and penetrated the muscularis in some parts (Figure 6B,C; Table 2). Granulomas composed of one or more *Schistosoma* eggs surrounded by leucocyte aggregates, eosinophils, plasma cells, macrophages, lymphocytes, and neutrophils distribute the intestinal structure and damage crypts and villi, thick submucosal walls, cellular infiltration in the lamina propria, and edema in the mucosa, indicating the occurrence of severe chronic inflammation during infection. The proliferation of fibrous tissue around the ova leads to the formation of a thick fibrous wall extending through the different layers of the intestine. The intestinal sections from all treated mice (PZQ, TOR, or PZQ+TOR) showed a noticeable amelioration of the intestinal layer structure with a significant decline (*p* < 0.05) in the fibrocellular granuloma diameter (40.55 ± 10.81 μm, 36.39 ± 10.36 μm, 26.27 ± 7.18 μm, respectively) compared with G2, the infected group without treatment (Figure 6D–F; Table 2). The greatest improvement was obtained in mice treated with both PZQ and TOR. Masson’s trichrome staining revealed collagen fiber deposition in the intestinal layers of the different groups, as indicated by the green-bluish staining (Figure 7A–F; Table 2). Highly fibrous proliferation and extension around the granulomas were detected in the infected untreated group, while dual treatment (G5; PZQ+TOR) resulted in minimal fibrous deposition compared to that in the G2-infected untreated group.

### 3.4. Cellular Immune Response

**Figure 8:**
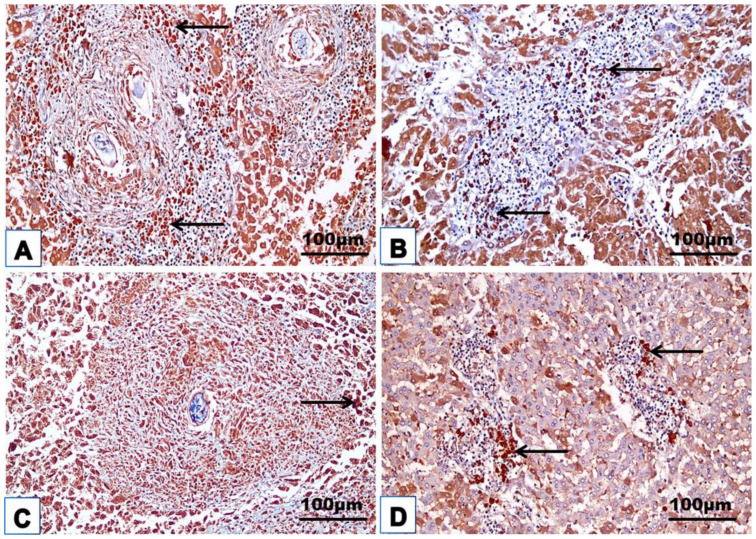
Representative photomicrographs of the immunohistochemical hepatic sections (n = 5) of the infected mice without treatment (**A**), with PZQ monotherapy (**B**), with TOR monotherapy (**C**), and with PZQ+TOR dual therapy (**D**), demonstrating positive brown CD3^+^ reactions (arrows).

**Figure 9:**
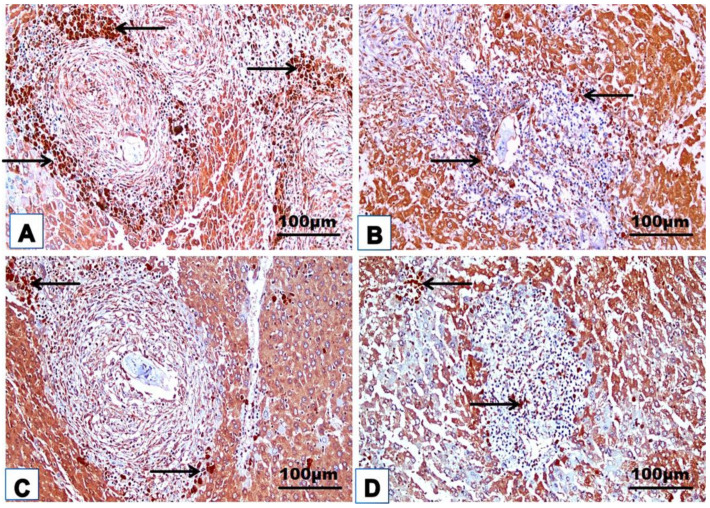
Representative photomicrographs of the immunohistochemical hepatic sections (n = 5) of infected mice without treatment (**A**), with PZQ monotherapy (**B**), with TOR monotherapy (**C**), and with PZQ+TOR dual therapy (**D**), demonstrating positive brown CD20^+^ reactions (arrows).

**Figure 10:**
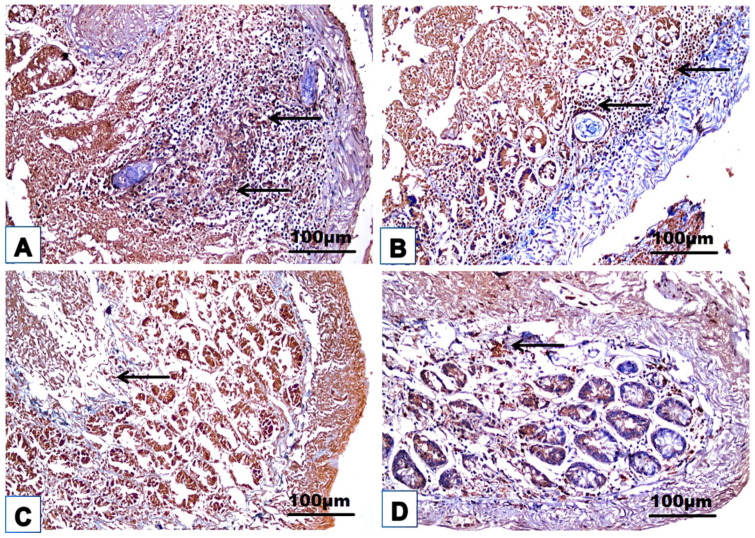
Representative photomicrographs of the immunohistochemical intestinal sections (n = 5) of infected mice without treatment (**A**), with PZQ monotherapy (**B**), with TOR monotherapy (**C**), and with PZQ+TOR dual therapy (**D**), demonstrating positive brown CD3^+^ reactions (arrows).

**Figure 11:**
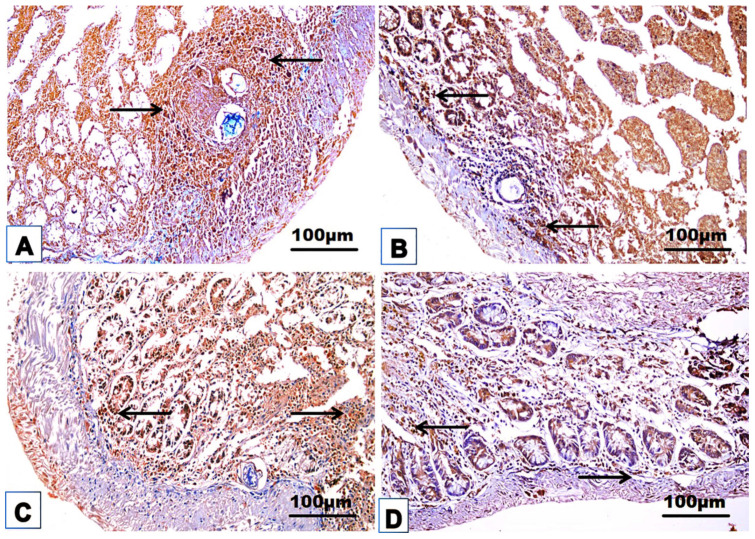
Representative photomicrographs of the immunohistochemical intestinal sections (n = 5) of infected mice without treatment (**A**), with PZQ monotherapy (**B**), with TOR monotherapy (**C**), and with PZQ+TOR dual therapy (**D**), demonstrating positive brown CD20^+^ reactions (arrows).

**Figure 12:**
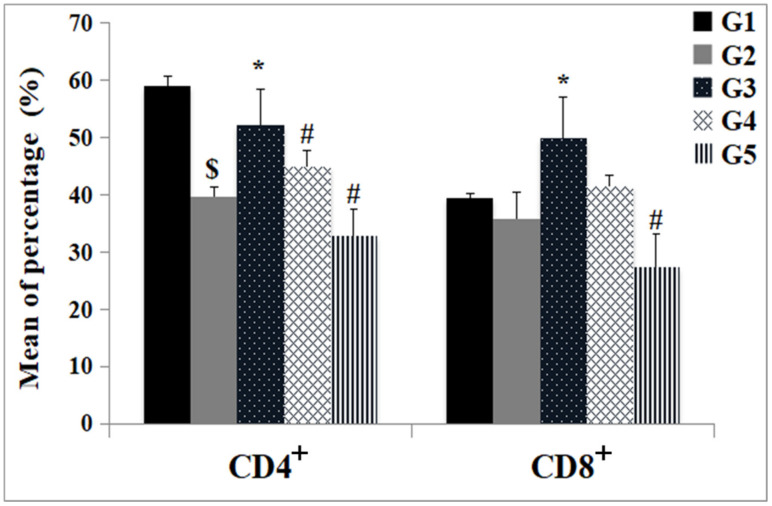
Histogram of the CD4^+^ and CD8^+^ blood phenotypic analysis after different therapies. The data are expressed as the means ± SD of the following groups (n = 3): the uninfected control group (G1), the infected untreated group (G2), the PZQ monotherapy group (G3), the TOR monotherapy group (G4), and the PZQ+TOR dual therapy group (G5). The significant (*p* < 0.05) differences are (*) compared to G2, (#) against G3, and ($) compared with G1.

The changes in the cellular immune response are shown in Table 3 and Figure 8, Figure 9, Figure 10, Figure 11 and Figure 12. Immunohistochemical staining was used to detect CD3^+^ and CD20^+^ lymphocytes in the hepatic and intestinal tissues (Table 3, Figure 8, Figure 9, Figure 10 and Figure 11), and flow cytometry was used to detect CD4^+^ and CD8^+^ cells in the blood (Figure 12).

#### 3.4.1. Immunohistochemical Examinations

CD3^+^ and CD20^+^ are common markers used to distinguish T- and B-lymphocytes infiltration, respectively. Immunohistochemistry revealed the immunoreaction of CD3^+^ and CD20^+^ as evidence of cellular infiltration into the liver and intestine of infected untreated animals, which exhibited high CD3^+^ and CD20^+^ expression in the liver and intestine (Figure 8A, Figure 9A, Figure 10A and Figure 11A; Table 3). Monotherapy of infected mice with PZQ or TOR resulted in moderate CD3^+^ and CD20^+^ expression in hepatic and intestinal sections (Figure 8B,C, Figure 9B,C, Figure 10B,C and Figure 11B,C) and a significant decrease in CD3^+^ and CD20^+^ expression compared to that in infected animals without treatment (Table 3). The dual therapy of PZQ and TOR resulted in CD3^+^ and CD20^+^ very low expression in the hepatic and intestinal sections (Figure 8D, Figure 9D, Figure 10D and Figure 11D), which was significantly the lowest compared to the infected untreated mice (Table 3).

#### 3.4.2. Subtyping of Immune Cells

An obvious decrease in the percentage of CD4^+^ and CD8^+^ cells was detected in the blood of the infected untreated group (G2) compared with the control group (G1) (Figure 12). Monotherapy with PZQ alone (G3) led to a significant increase in the percentage of CD4^+^ and CD8^+^ cells compared with that in G2. No significant change in the percentage of CD4^+^ or CD8^+^ cells was detected after TOR monotherapy (G4) compared with that in infected mice (G2). The combination of PZQ and TOR dual therapy (G5) decreased the number of CD4^+^ and CD8^+^ T cells compared to that in PZQ-treated mice (G2) (Figure 12).

### 3.5. Immune Response and Antioxidant Status

**Figure 13:**
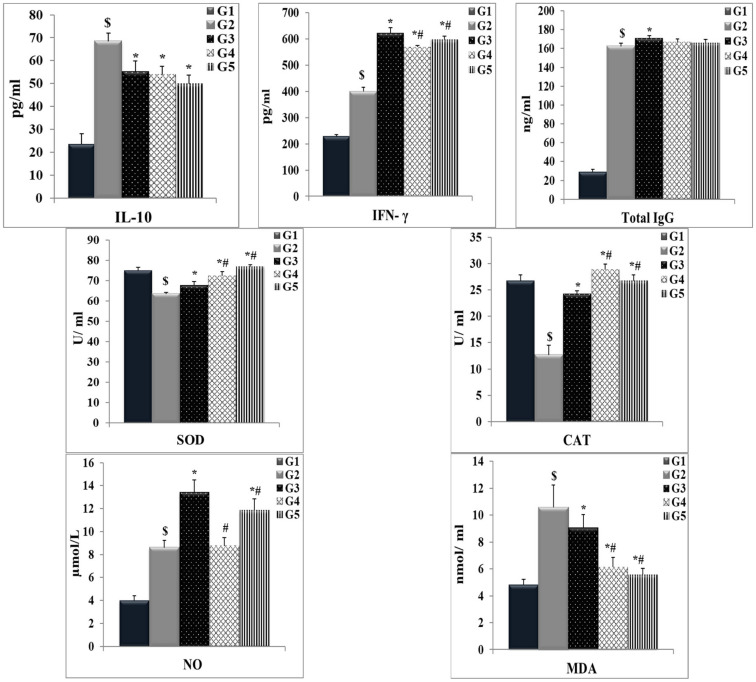
Histograms of the immune response and antioxidant status after different therapies. The data are expressed as the means ± SD of the following groups (n = 4): the uninfected control group (G1), the infected untreated group (G2), the PZQ monotherapy group (G3), the TOR monotherapy group (G4), and the PZQ+TOR dual therapy group (G5). Abbreviations: total immunoglobulin G (Total IgG), interleukin 10 (IL-10), interferon-gamma (IFN-γ), nitric oxide (NO), malondialdehyde (MDA), superoxide dismutase (SOD), and catalase (CAT). A significant (*p* < 0.05) difference is shown as (*) compared to G2, (#) against G3, and ($) compared with G1.

The histograms in Figure 13 show a significant increase (*p* < 0.05) in the serum total IgG, IL-10, IFN-γ, MDA, and NO levels and a significant decrease (*p* < 0.05) in the SOD and CAT activities in the infected mice without therapy (G2) compared with those in the uninfected control group (G1). All treatments (PZQ, TOR, and PZQ+TOR) elevated total IgG, IFN-γ, NO, SOD, and CAT, accompanied by an observable reduction in IL-10 and MDA compared with those in the infected mice without therapy (G2); the highest total IgG, IFN-γ, and NO levels were detected in the PZQ-treated group (G3). However, TOR monotherapy alone (G4) and dual therapy with PZQ+TOR (G5) resulted in a significant decrease in the IL-10 concentration compared with that in infected untreated animals. Oral administration of TOR alone enhanced NO production compared with that in the control group (G1) but did not significantly increase NO production compared with that in the infected group (G2). In addition, compared with PZQ monotherapy, dual therapy with PZQ and TOR slightly decreased the NO concentration. Almost all examined parameters from the PZQ and TOR dual-treatment groups were comparable to those of the normal control group.

## 4. Discussion

Bilharziasis is a common tropical parasitic disease that causes various clinical manifestations, and the main schistosomiasis therapy until now has been dependent on PZQ. The lower solubility of PZQ in water causes its lower absorption through the intestine, resulting in the production of a less potent compound, and its long-term use has led to the appearance of drug-resistant *Schistosoma* strains, so PZQ is unsuitable for therapy in endemic areas [12,13]. The serious side effects of PZQ and its global resistance have led to the search for bio-active natural plants that can be used as unconventional anti-parasitic agents. Thus, the present study evaluated the therapeutic effects of TOR alone or in combination with PZQ on mice infected with *S. mansoni*. The present study results suggested that TOR could be a potent source of natural antioxidants because of its high phenolic and flavonoid contents and its remarkable DPPH scavenging effects. TOR supplements could be significantly effective in preventing various harmful disorders due to their phytochemical contents. Dandelion consists of multiple flavonoids, polyphenols, alkaloids, carotenoids, glycosides, sterols, reducing sugars, and terpenes, which are used to remove the harmful action of free radicals [16,38]. These findings align with those of Xue et al. [39], who attributed the suppressive effects of dandelion on oxidative stress and inflammation in the intestinal epithelium to its chemical components. The therapeutic effects of many plants are attributed to their antioxidant potential [40,41]. The bio-active contents of phytochemicals are the major factor that controls the antioxidant mechanism involved in reactions between the free radicals in the solution of DPPH and the reactive species in the plant extract, such as phenolics and flavonoids [42,43]. The present study revealed that *S. mansoni* infection resulted in the upregulation of oxidative stress markers (MDA and NO) and the depletion of antioxidant enzymes (SOD and CAT). TOR supplementation with or without PZQ prevented the increase in MDA and ameliorated the decrease in antioxidant activity in the blood. The present findings clarified that TOR is a beneficial source of natural antioxidants and can be used to protect against various disorders related to oxidative stress. Antioxidant enzymes play a critical role in the intracellular defense against oxidative stress [44]. The daily administration of 200 mg TOR extract/kg to rats with liver and testicle injuries for 14 consecutive days significantly reduced the MDA level and increased the activity of CAT, SOD, GSH, and Gpx [15]. NO produced by macrophages stimulated the current infected and treated groups, which may generate antifibrotic substances around the hepatic and intestinal granulomas, causing a reduction in granuloma size. These findings align with Lebu et al. [45], who reported that immune responses against helminth infections modulate the host immune response to other antigens, decreasing chronic inflammation and tissue injury. Immune system disturbances are associated with bilharziasis-induced liver fibrosis through the production of different immunomodulatory agents with beneficial effects [46]. NO is the key effector molecule of activated macrophages involved in controlling infection by *S. mansoni* [47]. The current data align with Eid et al. [48] who reported an increase in the level of NO in the liver in the PZQ-monotherapy group. They attributed this increase to immune system activation via an increase in the IFN-γ level, which stimulates macrophages to produce NO and inflammatory mediators.

The current mono and dual therapies revealed a significant reduction in the total worm burden, an increase in the dead ova percentage, and the oogram pattern alterations recorded in infected untreated mice. Dual therapy of PZQ+TOR resulted in the most significant anti-parasitic effects. Moreover, different therapies caused severe peeling and rupture of the tubercles, the loss of some spines, and marked disturbances in the direction of the spines in the anterior region of the oral and ventral sucker, indicating that TOR itself has an anthelminthic effect by destroying the adult worm surface. This ability of TOR can be correlated with its antioxidant, anti-inflammatory, anti-parasitic, and anti-fibrotic properties, which may be attributed to its chemical components. These anti-parasitic effects of TOR against parasitic infection are consistent with those of Khan et al. [49], who reported that whole-plant dandelion extract initiated an immune response against the cattle parasite “*Rhipicephalus microplus*”. Dedić et al. [50] reported that the ethanolic extract of dandelion had various flavonoid and polyphenol compounds that are known to provide multiple health benefits, such as antioxidant, anti-inflammatory, antidepressant, and anti-carcinogenic effects. Terpene and sesquiterpene lactones, which are dandelion components, cause complete damage to the worm tegument and, ultimately, the death of the worms in schistosomiasis mice [51]. Daily administration of whole-plant dandelion extract (500 mg/kg bw) to rats suffering from liver fibrosis significantly increased antioxidant activity. It decreased hepatic oxidative stress markers [8]. Therapy using whole-plant dandelion extract against adult female *Rhipicephalus microplus* significantly reduced egg production, increased larval mortality, and increased the percentage of oviposition inhibition in adult female ticks [49]. The tegumental, ventral, and oral sucker damage caused by PZQ and TOR results in the loss of the worm’s ability to attach to blood vessel walls, consequently impairing or completely preventing the absorption of nutrient materials from the blood and destroying of the worm defense system, which leads to worm death. Similarly, Praziquantel and Nitazoxanide disrupted *Schistosoma* worms via mediating immune responses in mice [52].

The light microscopic examinations in this study revealed several histological alterations in hepatic and intestinal sections of untreated bilharziasis mice. In this study, mono and dual therapies caused a reduction in the granuloma diameter and cellularity, and collagen fibrous deposition, compared with those in the infected untreated group by regulating the immune response against the parasite through the immunomodulation of CD3^+^ and CD20^+^ and CD4^+^ and CD8^+^ lymphocytes in the different examined tissues. Excessive collagen deposition in bilharziasis obstructs normal hepatic and intestinal architectures. It gradually damages their functions, promoting the progression of the immune response and infiltration of inflammatory cells. Furthermore, the administration of TOR with or without PZQ disrupted liver injury, decreased collagen deposition, and regulated cellular immunity. This hepatoprotection may be attributed to oxidative stress suppression and the regulation of inflammatory signaling pathways related to the antioxidant constituents of dandelion. Daily whole-plant dandelion administration to fibrotic liver rats significantly improved their histologic liver architecture, reduced collagen fiber deposition, and decreased the immune protein expression of α-smooth muscle actin types 1 and 3 collagen [8]. Wirngo et al. [53] concluded that the anti-fibrotic effect of dandelion is related to its antioxidant components. Domitrović et al. [22] reported that the administration of dandelion extract to fibrotic liver mice caused a marked decrease in hepatic fibrous deposition and restored the histoarchitecture of the liver. Excess collagen fiber deposition is a characteristic feature of the fibrotic liver [54]. The current data agree with those of El-Sayed et al. [55], who concluded that PZQ has a regulatory influence on cellular immune responses, increasing CD8^+^ cells and reducing hepatic granuloma size in bilharziasis. Purified lung-stage schistosomula antigen immunization markedly decreased the worm burden, egg load, granuloma diameter, and collagen deposition and ameliorated histopathological alterations in the lungs and liver of *S. mansoni* model mice by increasing the levels of specific immunoglobulins [56].

Oxidative stress, hepatic and intestinal injuries, and fibrosis in infected untreated animals involve cellular infiltration and inflammatory marker expression, revealing the major role of hepatic and intestinal degradation and fibrosis regulation caused by bilharziasis. These findings are consistent with Vacca and Le Gros [57], who concluded that an increase in proinflammatory cytokines characterizes the host’s immune response against parasitic infections. In contrast, all current therapies demonstrated significantly increased levels of Total IgG and INF-γ (Th1 cytokine) accompanied by a significant decrease in IL-10 (Th2 cytokine) compared with those in the infected untreated group, thus confirming the anti-inflammatory effect of TOR. These findings are consistent with those of Sharaf et al. [58], who concluded that IL-10 and IFN-γ are influential regulators of the immuno-inflammatory response and are necessary to prevent severe pathology; indeed, PZQ therapy induced a significant increase in the level of IFN-γ in murine models. The current monotherapy of infected mice with PZQ or TOR significantly decreased the level of IL-10 compared to that in the infected animals without treatment, while dual therapy with PZQ and TOR significantly decreased the IL-10 level. These data agree with those of Madbouly et al. [59], who reported that PZQ monotherapy in *S. mansoni*-infected mice caused a significant decrease in the level of IL-10 compared with that in the infected untreated group, indicating that PZQ has immunosuppressive effects. The present study showed that TOR monotherapy was associated with a shift from Th2 to Th1. These findings are consistent with those of El-Sisi et al. [46], who reported that various types of flavonoids significantly increase the activity of T helper lymphocytes, increase IL-2 and IFN-γ levels, and activate macrophages. Therefore, the immunostimulatory action induced by PZQ or TOR may be attributed to several cellular and humoral antibody-mediated immune mechanisms against infections.

## 5. Conclusions

Dandelion root extract supplements have an anthelminthic ability and improve the therapeutic impacts of praziquantel in a bilharziasis mouse model by enhancing the antioxidant, anti-fibrotic, and immunomodulatory effects against the examined infection. Mono or dual treatment with dandelion revealed noticeable topographical SEM disruptions of male adult worms, a critical reduction in ova count and worm burden, hepatic and intestinal histological changes with markable depletion of fibrinous granuloma diameter, positive impacts on cellular immune responses (CD3^+^, CD20^+^, CD4^+^, and CD8^+^ cells), oxidative stress (MDA and NO), and interleukin-10, and upregulation of total immunoglobulin G, interferon-gamma, and antioxidant enzymes (SOD and CAT), in comparison with infected untreated mice. Further studies are required to detect the molecular, genomic, transcriptomic, and proteomic reasons behind the exact healing/relieving mechanisms of the TOR against the *Schistosoma* infection. Further studies will be required in order to detect the clinical dose of TOR against schistosomiasis in humans.

## Figures and Tables

**Table 1:** (**a**–**c**). Effects of monotherapy and dual therapy on the worm burden, ova count, and oogram pattern of *S. mansoni*-infected mice.

Group	a—Worm burden					
	Male	Female	Couple		Total	Reduction (%)
G2	3.20 ± 0.84	1.00 ± 0.00	6.80 ± 0.84		11.00 ± 1.00	-
G3	1.40 ± 0.55 *	1.00 ± 0.00	2.00 ± 1.00 *		4.4 ± 1.14 *	60.64%
G4	1.20 ± 0.45 *	0.00	2.80 ± 0.84 *		4.00 ± 1.00 *	63.64%
G5	1.80 ± 0.84 *	0.00	1.60 ± 0.55 *		3.40 ± 0.55 *	69.10%
**Group**	**b—Ova Count**					
	**Liver**		**Reduction (%)**		**Intestine**	**Reduction (%)**
G2	4957.66 ± 371.20		00.00%		7845.20 ± 210.03	-
G3	931.73 ± 80.53 *		81.21%		1152.33 ± 118.88 *	85.31%
G4	1362.26 ± 223.28 *^#^		72.52%		2506.60 ± 103.82 *^#^	68.05%
G5	906.59 ± 116.39 *		81.71%		1030.93 ± 91.35 *	86.86%
**Group**	**c—Oogram Pattern**					
	**Liver**			**Intestine**		
	**Mature**	**Immature**	**Dead**	**Mature**	**Immature**	**Dead**
G2	78.06 ± 0.64	16.80 ± 0.38	5.13 ± 0.50	71.53 ± 1.07	20.93 ± 1.52	7.53 ± 0.61
G3	15.26 ± 1.59 *	21.86 ± 0.93	62.87 ± 1.61 *	8.66 ± 1.03 *	22.53 ± 1.39	68.80 ± 2.27 *
G4	23.80 ± 1.39 *^#^	16.40 ± 0.43 ^#^	59.80 ± 1.07 *^#^	16.00 ± 2.26 *^#^	18.47 ± 2.66 ^#^	65.53 ± 3.76 *
G5	14.13 ± 1.59 *	15.33 ± 0.94 *^#^	70.53 ± 1.97 *^#^	9.53 ± 0.96 *	13.33 ± 1.54 *^#^	77.13 ± 1.44 *^#^

The data are presented as the mean ± SD of the following groups (n = 5): infected untreated group (G2), PZQ monotherapy group (G3), TOR monotherapy group (G4), and PZQ+TOR dual therapy group (G5). A significant (*p* < 0.05) difference is indicated by (*) compared to G2 and (#) compared to G3.

**Table 2:** The different groups’ granuloma diameters and positive expression of collagen fibers in the liver and intestine.

Group	Liver		Intestine	
	Granuloma Diameter (µm)	Collagen Fibers %	Granuloma Diameter (µm)	Collagen Fibers %
G1	Nil	1.20 ± 0.58	Nil	0.51 ± 0.22
G2	152.93 ± 37.43	14.77 ± 4.14 ^$^	62.62 ± 2.08	7.71 ± 1.11 ^$^
G3	90.14 ± 17.83 *	5.73 ± 2.21 *	40.55 ± 10.81 *	4.87 ± 0.85 *
G4	80.56 ± 21.19 *	5.01 ± 1.46 *	36.39 ± 10.36 *	3.86 ± 1.20 *
G5	38.97 ±10.25 *^#^	1.84 ± 0.90 *^#^	26.27 ± 7.18 *^#^	1.59 ± 0.68 *^#^

The data are expressed as the mean ± SD of the following groups: uninfected control (G1), infected untreated (G2), PZQ-monotherapy (G3), TOR-monotherapy (G4), and PZQ+TOR-dual therapy (G5), forty granuloma from different sections of each group (n = 5). A significant (*p* < 0.05) difference is indicated by (*) compared with G2, (#) against G3, and ($) compared with G1.

**Table 3:** The percentages (%) CD3^+^ and CD20^+^ cells expression in hepatic and intestinal sections after different therapies.

Group	CD3^+^ %		CD20^+^ %	
	Liver	Intestine	Liver	Intestine
G2	5.50 ± 1.64	11.42 ± 2.32	8.70 ± 0.72	6.39 ± 2.31
G3	2.10 ± 0.35 *	4.13 ± 1.26 *	4.59 ± 0.98 *	4.41 ± 1.33 *
G4	2.46 ± 0.67 *	3.24 ± 0.98 *	2.34 ± 0.79 *^#^	2.70 ± 0.85 *
G5	1.64 ± 0.43 *	1.75 ± 0.55 *^#^	1.58 ± 0.46 *^#^	1.63 ± 0.49 *^#^

The data are expressed as the mean ± SD and represent the percentages of CD3^+^ and CD20^+^ immune cells in the different groups (n= 5): the infected untreated group (G2), the PZQ monotherapy group (G3), the TOR monotherapy group (G4), and the PZQ+TOR dual therapy group (G5). A significant (*p* < 0.05) difference is indicated by (*) compared to G2 and (#) compared to G3.

## Data Availability

All data used in this study are included in this published article.
